# Supplementary material for: Gaps between Open Science activities and actual recognition systems: Insights from an international survey
Source: PLoS One. 2024 Dec 16;19(12):e0315632. doi: 10.1371/journal.pone.0315632 (PMC11649118; doi:10.1371/journal.pone.0315632)
Supplement: S1 File — (PDF) [file pone.0315632.s001.pdf]

RDA- SHARC Open Science survey

The goal of this survey is to identify perceptions and expectations of various research communities regarding how Open Science activities are (or should be) taken into consideration and rewarded. The results will help with future recommendations towards various stakeholder groups involved in research evaluation.

This survey is being conducted as part of the SHAring Rewards & Credit (SHARC) interest group within Research Data Alliance (RDA).

Preliminary survey results will be presented and discussed during the RDA 19th Plenary meeting , as part of the International Data Week 2022 taking place 20-23 June 2022.

(The average completion time is about 15 min)

There are 19 questions in this survey.

Section 1

In this section, we want to know how familiar you are with Open Science and if you are involved in related activities.

Are you familiar with Open Science? *

Please choose **only one** of the following:

- ◯ Yes
- ◯ No

Are you involved in some of the following Open Science activities such as: *

Check all that apply

Please choose **all** that apply:

- ☐ sharing a research manuscript as a preprint
- ☐ publishing a paper or monograph/book as Open Access
- ☐ preregistration of the study design, methods, hypothesis, etc. prior to commencing the research
- ☐ open or FAIR (Findable, Accessible, Interoperable, Reusable) data management and sharing (for research data, software, models, algorithms, workflows etc.)
- ☐ participation in open peer review (being reviewed or the reviewer)
- ☐ outreach and public engagement in science, including citizen (or community) science
- ☐ collaboration via virtual research environments/virtual laboratories
- ☐ none of the above

Are you involved in any other Open Science activities that you would like to mention? If yes, please specify.

Please write your answer here:

Are you familiar with the FAIR (Findable, Accessible, Interoperable, and Reusable) principles (defined here)? *

Please choose **only one** of the following:

- Yes
- No

Are you involved in some steps of data FAIRification process such as those referred to here ?

Only answer this question if the following conditions are met:
((G00Q05.NAOK == 'Y'))

Please choose **only one** of the following:

- Yes
- No

Specify which ones?

Only answer this question if the following conditions are met:
((G00Q06.NAOK == 'Y'))

Please write your answer here:

Does your institute /organisation have policies on various Open Science activities?

Please choose **only one** of the following:

- ◯ Yes
- ◯ No

Could you please specify briefly what kind of policy and provide a link to any relevant one, if possible?

Only answer this question if the following conditions are met:
((G01Q11.NAOK == 'Y'))

Please write your answer here:

Section 2

In this section, we want to know your ranked priorities with regard to rewarding Open Science activities.

Could you please specify to what extent you feel the following activities should be credited / rewarded? Rewards may include career promotion, grants/funding/prizes, gained credits in a research evaluation procedure, authorship/ contributorship, increased academic visibility.

Please choose the appropriate response for each item:

	Definitely	Very Probably	Possibly	Probably Not	Definitely Not	No opinion
Sharing a research manuscript as a preprint	○	○	○	○	○	○
Publishing a paper or monograph/book as Open Access (‘Gold’ or ‘Green’)	○	○	○	○	○	○
Preregistration of the study design, methods, hypothesis, etc. prior to commencing the research	○	○	○	○	○	○
Open or FAIR (Findable, Accessible, Interoperable, Reusable) data management and sharing (for research data, software, models, algorithms, workflows etc.)	○	○	○	○	○	○
Participation in open peer review (being reviewed or the reviewer)	○	○	○	○	○	○
Participation in public engagement, including citizen (or community) science	○	○	○	○	○	○

	Definitely	Very Probably	Possibly	Probably Not	Definitely Not	No opinion
**Collaboration via virtual research environments/virtual laboratories**	◯	◯	◯	◯	◯	◯

Section 3

In this section, we want to know if your affiliated organisation has any rewards for Open Science activities.

Does your institute/organisation have any initiative or tool which gives credits/rewards for Open Science activities?

*

Please choose **only one** of the following:

- ◯ Yes
- ◯ No

You’re welcome to point here to any document/link/contact to know more about it.

Only answer this question if the following conditions are met:
((G03Q08.NAOK == 'Y'))

Please write your answer here:

Section 4

In this section, we want you to share your opinion on ideal rewarding paths for Open Science activities.

How would you want the previously mentioned Open Science activities to be rewarded ?

Please write your answer here:

eg, rewards can be related to career promotion, grants/funding/prizes, visibility gained credits in a research evaluation procedure, authorship/ contributorship, increased academic or other ways you may suggest)

Section 5

In the final section, we will ask a few questions about your background.

Main position/job title: which of the following are you now? *

❗ Choose one of the following answers
Please choose **only one** of the following:

- ◯ Professor
- ◯ Researcher
- ◯ Postdoc
- ◯ Graduate student (Master, PhD)
- ◯ Software engineer
- ◯ Data specialist
- ◯ Policy officer
- ◯ Other, please specify in the comment box

Make a comment on your choice here:

Please select the type of organisation you are primarily affiliated with: *

Choose one of the following answers

Please choose **only one** of the following:

- University
- Research institute
- NGO / NPO
- Government agency
- International organisation
- Publisher (e.g., scholarly societies)
- Other, please specify

Make a comment on your choice here:

Which country do you work in? *

Choose one of the following answers
Please choose **only one** of the following:

- ◯ Afghanistan
- ◯ Africa Eastern and Southern
- ◯ Africa Western and Central
- ◯ Albania
- ◯ Algeria
- ◯ American Samoa
- ◯ Andorra
- ◯ Angola
- ◯ Antigua and Barbuda
- ◯ Arab World
- ◯ Argentina
- ◯ Armenia
- ◯ Aruba
- ◯ Australia
- ◯ Austria
- ◯ Azerbaijan
- ◯ Bahamas, The
- ◯ Bahrain
- ◯ Bangladesh
- ◯ Barbados
- ◯ Belarus
- ◯ Belgium
- ◯ Belize
- ◯ Benin
- ◯ Bermuda
- ◯ Bhutan

- ◯ Bolivia
- ◯ Bosnia and Herzegovina
- ◯ Botswana
- ◯ Brazil
- ◯ British Virgin Islands
- ◯ Brunei Darussalam
- ◯ Bulgaria
- ◯ Burkina Faso
- ◯ Burundi
- ◯ Cape Verde
- ◯ Cambodia
- ◯ Cameroon
- ◯ Canada
- ◯ Caribbean small states
- ◯ Cayman Islands
- ◯ Central African Republic
- ◯ Central Europe and the Baltics
- ◯ Chad
- ◯ Channel Islands
- ◯ Chile
- ◯ China
- ◯ Colombia
- ◯ Comoros
- ◯ Congo, Dem. Rep.
- ◯ Congo, Rep.
- ◯ Costa Rica
- ◯ Ivory Coast
- ◯ Croatia
- ◯ Cuba
- ◯ Curaçao

- ◯ Cyprus
- ◯ Czech Republic
- ◯ Denmark
- ◯ Djibouti
- ◯ Dominica
- ◯ Dominican Republic
- ◯ Ecuador
- ◯ Egypt, Arab Rep.
- ◯ El Salvador
- ◯ Equatorial Guinea
- ◯ Eritrea
- ◯ Estonia
- ◯ Eswatini
- ◯ Ethiopia
- ◯ Faroe Islands
- ◯ Fiji
- ◯ Finland
- ◯ France
- ◯ Gabon
- ◯ Gambia, The
- ◯ Georgia
- ◯ Germany
- ◯ Ghana
- ◯ Gibraltar
- ◯ Greece
- ◯ Greenland
- ◯ Grenada
- ◯ Guam
- ◯ Guatemala
- ◯ Guinea

- ◯ Guinea-Bissau
- ◯ Guyana
- ◯ Haiti
- ◯ Honduras
- ◯ Hong Kong SAR, China
- ◯ Hungary
- ◯ Iceland
- ◯ India
- ◯ Indonesia
- ◯ Iran, Islamic Rep.
- ◯ Iraq
- ◯ Ireland
- ◯ Isle of Man
- ◯ Israel
- ◯ Italy
- ◯ Jamaica
- ◯ Japan
- ◯ Jordan
- ◯ Kazakhstan
- ◯ Kenya
- ◯ Kiribati
- ◯ Korea, Dem. People's Rep.
- ◯ Korea, Rep.
- ◯ Kosovo
- ◯ Kuwait
- ◯ Kyrgyz Republic
- ◯ Lao PDR
- ◯ Latvia
- ◯ Lebanon
- ◯ Lesotho

- ◯ Liberia
- ◯ Libya
- ◯ Liechtenstein
- ◯ Lithuania
- ◯ Luxembourg
- ◯ Macao SAR, China
- ◯ Madagascar
- ◯ Malawi
- ◯ Malaysia
- ◯ Maldives
- ◯ Mali
- ◯ Malta
- ◯ Marshall Islands
- ◯ Mauritania
- ◯ Mauritius
- ◯ Mexico
- ◯ Micronesia, Fed. Sts.
- ◯ Moldova
- ◯ Monaco
- ◯ Mongolia
- ◯ Montenegro
- ◯ Morocco
- ◯ Mozambique
- ◯ Myanmar
- ◯ Namibia
- ◯ Nauru
- ◯ Nepal
- ◯ Netherlands
- ◯ New Caledonia
- ◯ New Zealand

- ◯ Nicaragua
- ◯ Niger
- ◯ Nigeria
- ◯ North Macedonia
- ◯ Northern Mariana Islands
- ◯ Norway
- ◯ Oman
- ◯ Pakistan
- ◯ Palau
- ◯ Panama
- ◯ Papua New Guinea
- ◯ Paraguay
- ◯ Peru
- ◯ Philippines
- ◯ Poland
- ◯ Portugal
- ◯ Puerto Rico
- ◯ Qatar
- ◯ Romania
- ◯ Russian Federation
- ◯ Rwanda
- ◯ Samoa
- ◯ San Marino
- ◯ Sao Tome and Principe
- ◯ Saudi Arabia
- ◯ Senegal
- ◯ Serbia
- ◯ Seychelles
- ◯ Sierra Leone
- ◯ Singapore

- ◯ Slovak Republic
- ◯ Slovenia
- ◯ Solomon Islands
- ◯ Somalia
- ◯ South Africa Rep.
- ◯ South Sudan Rep.
- ◯ Spain
- ◯ Sri Lanka
- ◯ St. Kitts and Nevis
- ◯ St. Lucia
- ◯ St. Vincent and the Grenadines
- ◯ Sudan
- ◯ Suriname
- ◯ Sweden
- ◯ Switzerland
- ◯ Syrian Arab Republic
- ◯ Tajikistan
- ◯ Tanzania
- ◯ Thailand
- ◯ Timor-Leste
- ◯ Togo
- ◯ Tonga
- ◯ Trinidad and Tobago Rep.
- ◯ Tunisia
- ◯ Turkey
- ◯ Turkmenistan
- ◯ Tuvalu
- ◯ Uganda
- ◯ Ukraine
- ◯ United Arab Emirates

- United Kingdom
- United States
- Uruguay
- Uzbekistan
- Vanuatu
- Venezuela, RB
- Vietnam
- Yemen, Rep.
- Zambia
- Zimbabwe
-

What are your main disciplinary fields (up to 3; e.g., computer science, physics, biology, medicine)? *

Please write your answer here:

How long have you been working in your field?

Choose one of the following answers

Please choose **only one** of the following:

- < 5 y
- 5-10 y
- 10-20 y
- > 20 y

Would you have anything else you think is relevant to mention?

Please write your answer here:

Your gender:

Please write your answer here:

Thank you very much for completing this questionnaire! In case of any questions/comments/suggestions arising with regard to this survey, feel free to contact one of us:

laurence.mabile@univ-tlse3.fr

anne.cambon-thomsen@univ-tlse3.fr

hanna.shmagun@kisti.re.kr

CErdmann@agu.org

grattarola@fzp.czu.cz

In addition, if you are excited to discover the preliminary outcomes of this study, you can attend the RDA 19th Plenary meeting or send us an email to request the results of the survey.

Submit your survey.
Thank you for completing this survey.
