# Supplementary material for: Gaps between Open Science activities and actual recognition systems: Insights from an international survey
Source: PLoS One. 2024 Dec 16;19(12):e0315632. doi: 10.1371/journal.pone.0315632 (PMC11649118; doi:10.1371/journal.pone.0315632)
Supplement: S2 File — (PDF) [file pone.0315632.s002.pdf]

Supporting Information S2

Full list of organisations used for the dissemination of survey questionnaire

- French National Centre for Scientific Research (French: Centre national de la recherche scientifique, CNRS) / Open Research Data Department (DDOR)
- University of Toulouse / Open Science Committee
- National Aeronautics and Space Administration (NASA) / Transform to Open Science (TOPS) Project Office
- PARSEC project (Building New Tools for Data Sharing and Reuse through a Transnational Investigation of the Socioeconomic Impacts of Protected Areas) team
- European Federation for Immunogenetics (EFI) community
- Korea Institute of Science and Technology Information (KISTI)
- Uruguayan Consortium of Biodiversity Data (Biodiversidata)
- Research Data Alliance United States (RDA-US)
- Australian Research Data Commons (ARDC)
- European Geosciences Union (EGU)
- European Organization for Nuclear Research (CERN)
- Aquatic Data Sciences
- Earth Science Information Partners (ESIP)
- European Southern Observatory (ESO)
- GO FAIR US office
- EarthCube (A Community Dedicated to the Advancement of Geoscience Research)
- Open Science MOOC
- Chan Zuckerberg Initiative (CZI)
- Science Research & Development Society (SRDS)
- OSC
- Framework for Open and Reproducible Research Training (FORRT)
- SciELO (Scientific Electronic Library Online)
- CODATA / International Data Policy Committee (IDPC)
- Electronic Information for Libraries (EIFL)
- Tallinn University of Technology
- Robert Gordon University
