# Supplementary material for: Gaps between Open Science activities and actual recognition systems: Insights from an international survey
Source: PLoS One. 2024 Dec 16;19(12):e0315632. doi: 10.1371/journal.pone.0315632 (PMC11649118; doi:10.1371/journal.pone.0315632)
Supplement: Attachment — Submitted filename: Rebuttal Letter_response to reviewers.pdf [file pone.0315632.s005.pdf]

RE: submission of the revised manuscript to PLOS ONE

Erum Shaikh

Academic Editor

PLOS ONE

We are pleased to submit our revised manuscript (PONE-D-24-06329R1), "Gaps between Open Science activities and actual recognition systems: Insights from an international survey" by Grattarola, Shmagun, et al., to PLOS ONE.

We are grateful to you and the reviewers for the diligent review work. We have made a concerted effort to address all the reviewers' comments and recommendations to enhance the quality of our work's communication.

The comments/suggestions offered by the referees have improved the manuscript. Specifically, we have:

- Included a statistical test to assess the reliability of our survey questions that used binary, Likert and ordinal scales, revealing an acceptable internal consistency (see the Methods section).
- Added an additional aspect to the limitations, as suggested by Reviewer 2, noting that the seven types of open science activities proposed in our survey are not exhaustive.

In addition to the above changes, all additional suggestions have been taken into account and addressed, and we now provide a full revision of our manuscript. Responses to all comments one by one are outlined in detail below.

Yours sincerely,

Hanna Shmagun, on behalf of the co-authors

**Comments**	**Response**
	**Academic Editor**
1. Please ensure that your manuscript meets PLOS ONE's style requirements, including those for file naming…	We made the revision to meet PLOS ONE's style requirements.
2. Please include a complete copy of PLOS' questionnaire on inclusivity in global research in your revised manuscript.	This questionnaire was completed and included as the Supporting Information S3 File. Additionally, we provided a reference in the Methods section: "Additional information regarding the ethical, cultural and scientific considerations specific to inclusivity in global research is included in the S3 File (Checklist)."
3. We note that the grant information you provided in the 'Funding Information' and 'Financial Disclosure' sections do not match. Please ensure that you provide the correct grant numbers for the awards you received for your study in the 'Funding Information' section.	
4. Please state what role the funders took in the study. If the funders had no role, please state: "The funders had no role in study design, data collection and analysis, decision to publish, or preparation of the manuscript." If this statement is not correct you must amend it as needed. Please include this amended Role of Funder statement in your cover letter; we will change the online submission form on your behalf.	We addressed this aspect in the following line.
5. … funding information should not appear in the Acknowledgments section or other areas of your manuscript. We will only publish funding information present in the Funding Statement section of the online submission form. Please remove any funding-related text from the manuscript and let us know how you would like to update your Funding Statement.	Funding-related text was removed from the Acknowledgement section of the manuscript. We would like to update the Funding Statement as follows: "This research received funding from the PARSEC Belmont Forum Collaborative Research Action on Science-Driven e-Infrastructures Innovation (SEI2018). Additionally, FG was funded by the European Union (ERC, BEAST, 101044740). HS received funding from the Korea Institute of Science and Technology Information (No. K-24-L01-C05-S01). ACT received funding from the European Union's Horizon 2020 research and innovation programme EOSC Future under grant agreement no 101017536 as domain ambassador. The funders had no role in study design, data collection and analysis, decision to publish, or preparation of the manuscript."
	**Reviewer 1**
As the authors state, the responses to this survey are not representative of a specific population, the lack of information on how many people it reaches makes it impossible to calculate the response rate, and according to the data provided and the institutions that participated, the results may be biased by the procedures and opinions of a population that is familiar with and participates in the OS.	We acknowledged this aspect in the limitations section.<br><br>"A main limitation of this study is that our survey sample is not representative of any specific defined population, but that which we were able to reach through the author's networks. While this approach was valuable for informing OS policy recommendations grounded in empirical information, it rendered cross-tabulation analyses irrelevant to this study. Despite the survey sample not being representative, as a cross-cultural group of authors we were able to crosswalk diverse perspectives on what rewards and incentives can mean in different countries and cultures. Thus, the findings of our study can contribute to OS theory and practice by suggesting OS rewards terminology and can inform a broad range of stakeholders, e.g., involved in research and innovation systems, in implementing OS rewarding schemes."

**Comments**	**Response**
in material and methods I do not see that the survey was previously tested to see the degree of difficulty and comprehension of the questions and answers. Nor do they mention whether the reliability of the survey questions was assessed, e.g. with the application of Cronbach's alpha (a high Cronbach's alpha values indicate that response values for each participant across a set of questions are consistent).	Relevant text was added to the Methods section. We calculated Cronbach's alpha to assess the reliability of the questions that used binary, Likert, and ordinal scales, revealing an acceptable internal consistency with a value of 0.71 (CI= 0.63, 0.76).
Regarding the open-ended responses, according to the results, there was not much participation, which limits their analysis, and they are barely mentioned in the text.	We received a robust response rate to the open-ended questions. However, the aggregated responses provided in the submitted S4 File do not include these open-ended responses. This exclusion was due to the fact that respondents often included many details, such as the names of their affiliations, which, when combined with other information, could potentially reveal their identities.<br>We clarified this aspect in a relevant sentence of the manuscript: "Aggregated responses can be found in the S4 File *(note that open-ended responses are excluded because they contain details that could be cross-referenced with other data sources, potentially leading to deanonymisation)*."
the fact that the survey was sent in several languages may make it difficult to interpret due to cultural and academic environment issues.	We conducted considerable work to ensure consistency across the survey's language versions. The translations into Korean and Spanish were specifically designed to better engage with local communities in Korea and Spanish-speaking countries such as Uruguay, where English might not be widely comfortable. Before distribution, the questionnaire was rigorously tested in each language by both the authors and their colleagues to ensure the clarity and comprehensibility of the questions; special attention was given to ensuring the accuracy of the translated versions of the questionnaire.
I recommend this article, not cited in this paper, which analyzes similar data but with more extension "Habits and perceptions regarding open science by researchers from Spanish institutions" https://doi.org/10.1371/journal.pone.0288313.	We cited the paper in the first paragraph of the Discussion.

Comments	Response
**Reviewer 2**	
A minor limitation might be that the questions on OS were not soo extensive, only 7 practices were asked (preprint, open access, preregistration, FAIR data, open peer review, citizen science, virtual labs) – and only these practices could be rated whether they should be more incentivized/rewarded. Maybe this could be briefly mentioned in the manuscript (e.g. under limitations)… Further criteria could be open source software, reproducibility (e.g. detailed methodologies), Registered Reports (I think it was not clarified whether it felt under preregistration?), open licensing, open policy, open educational resources, open science advocacy and community building, etc. – although the selected 7 criteria are definitely of high interest and relevant.	We added this aspect to the limitations.<br><br>Open source software is under the category "Open or FAIR data management and sharing (for research data, software, models, algorithms, workflows etc.)".<br><br>Registered Reports are under the category "Preregistration…".
Related to the title, I was wondering whether "Open Science policies" is the best name. The methods and results section says that "Open Science activities" or "OS practices" were rated regarding respondents' familiarity, preferences for OS activities, whether these activities are rewarded and should be rewarded. In my opinion, no "policies" (e.g. institutional) in the narrower sense were examined.	We amended the title according to the reviewer's suggestion as "Gaps between Open Science *activities* and actual recognition systems: Insights from an international survey".
There is a spelling error in the Introduction, it says MERRITT instead of MERRIT (line 52).	Corrected.
