# Supplementary material for: Gaps between Open Science activities and actual recognition systems: Insights from an international survey
Source: PLoS One. 2024 Dec 16;19(12):e0315632. doi: 10.1371/journal.pone.0315632 (PMC11649118; doi:10.1371/journal.pone.0315632)
Supplement: Attachment — Submitted filename: Response to reviewers comments.docx [file pone.0315632.s006.docx]

RE: Re-submission to PLOS ONE

Dr Alberto Baccini

Academic Editor

PLOS ONE

We are pleased to resubmit our revised manuscript (**PONE-D-24-06329R2**), “*Gaps between Open Science activities and actual recognition systems: Insights from an international survey*” to PLOS ONE.

The comments and suggestions offered by the editor and the referees have indeed helped improve the manuscript. The main concerns we have addressed are: 1) clarification of the snowball non-probability sampling method used and the limitations of a moderate, non-representative sample obtained through it, 2) explanation of the study's added value and why it is worth publishing despite the aforementioned limitations, 3) improvements to figures, such as Fig 1 (distribution of the respondents’ disciplines), Fig 3 (likert scale of proposed OS activities to get rewarded), and Fig 4 (preferences on types of rewards).

In addition to these, we have taken into account all additional suggestions, addressed them, and we now provide a full major revision of our manuscript. Detailed responses to all comments, one by one, are below.

Yours sincerely,

Hanna Shmagun, on behalf of the co-authors

**Response to the Academic Editor**

1) There is a first problem related to the power of the sample: the snowballing technique used is not clarified (how large is the initial group? How was the length of the survey decided? Why are 234 responses considered sufficient?). Obviously, given the low sample, data about single countries are simply unreliable. The abstract omits to disclose the tiny number of respondents and presents the paper as containing significant data for single countries.

> We can provide the survey’s completion rate, which is over 62% (see line 158 of the revised manuscript). However, we cannot provide a response rate – defined as the percentage of people who complete a survey out of the total number invited to participate – because the snowball sampling method we used made it impossible to estimate the total number of people who received the survey invitation. We acknowledge that our sample size doesn’t represent the broader population of the global scientific community at large or researchers from a particular field or region; however, this limitation is inherent and common to the non-probability sampling method we chose to use – snowball sampling. Achieving representation was not the goal when we were designing our survey, as we aimed to gather a variety of opinions from as many diverse regions and communities as possible, rather than focusing on a sample representing specific groups from just a few countries for example. We distributed the survey under the international umbrella of RDA to open science networks and major research organisations (e.g., NASA and AGU), which were particularly helpful regarding the survey’s distribution (see the list of communities to which we directly distributed our survey in the S2 File). This is reflected, for instance, in the number of countries represented (from all continents). However, despite our extensive efforts to reach diverse research communities from different countries, including offering our survey in three languages, we did not achieve a high number of responses. This may be because OS reward schemes are a relatively new and understudied topic, with limited awareness among researchers and institutions. Having said that, we ended up with sufficient data collected from the survey, which served as an input for developing recommendations for open science rewards and incentives (see Mabile et al, 2024; doi: 10.15497/RDA/000117).

All in all, we agree that the limitation of a moderate and non-representative sample size should have been more clearly presented in our manuscript and we have now made this more explicit in the Limitation section of the manuscript (see lines 295-304).

*“A main limitation of this study is that our survey sample is quite moderate and not representative of any specific defined population, rather, it consists of those we were able to reach through the authors’ networks using the snowball non-probability sampling method. Even though the survey was distributed to various research communities without the specific aim of targeting exclusively researchers engaged in OS, the final set of respondents were primarily individuals familiar with or active in OS, introducing a bias that may reflect the limited uptake of OS activities among many scholars. While the survey data collected through the snowball sampling approach was valuable for informing our later recommendations for OS rewards and incentives endorsed by the RDA community [25], it made the applicability of statistical cross-tabulation analyses irrelevant to this study”.*

In addition, we added the number of respondents in the abstract, as requested (line 42):

*“(...) a global survey was conducted in the framework of the Research Data Alliance, recruiting 230 participants from five continents and 37 countries”.*

Below, we would like to highlight why the responses gathered through this survey are meaningful for the purpose of our study and why this research is worth publishing, despite the previously mentioned limitation of a moderate and non-representative sample size.

- Rewards and incentives in OS is a novel and understudied topic. Thus, this was a pilot study and exploratory in nature. However, this study and its findings, including the proposed Terminology for Open Science Credits and Rewards, can encourage and lay the foundation for larger, more comprehensive quantitative studies with more generalisable results.
- The interest in this novel topic addressed in our study has been already demonstrated. Our study’s preprint (<https://osf.io/preprints/socarxiv/hru2x>) received an open review (<https://prereview.org/reviews/11234562>) and has been cited once (doi: 10.1002/ece3.11698), indicating that there are few sources presenting findings on this highly relevant topic.

2) The discussion of the bias introduced by selecting people in an RDA group devoted to work on “sharing rewards and credits” is completely inadequate. Moreover, the questionnaire assumes that OS/OA activities should be rewarded and avoids the upstream question of desirability of incentives for OS/OA. In my opinion, the survey cannot be considered as representing opinion of scholars, but only of a very small group of people already working on OS/OA rewarding. From this point of view, I think that the context and limitation of the survey should be more carefully highlighted in the text.

> The questionnaire assumes this because empirical studies on data sharing have indicated that recognition and credit can be major concerns of researchers to adopting open science practices (Dallmeier-Tiessen et al. 2014, doi: 10.1080/13614576.2014.883936; Bezuidenhout et al. 2018, doi: 10.1080/11287462.2018.1441780; Chawinga et al. 2019, doi: 10.1016/j.lisr.2019.04.004; Kim et al. 2015, doi: 10.3138/jsp.46.4.05). Moreover, although we did not explicitly ask whether open science should be incentivised or rewarded, we did provide respondents with the option to specify the extent to which they feel various proposed open science activities should be credited or rewarded, if at all. For each activity listed, respondents could choose a rating on a Likert scale ranging from “Definitely rewarded” to “Definitely not rewarded” (see the first question in Section 2 of the questionnaire, S1 File), indicating whether they believe certain activities should receive specific rewards or not. Additionally, in Section 4, we included an open-ended question about how these activities should be rewarded (188 of the respondents, 82%, expressed their thoughts), which also allowed respondents to express the view that specific rewards for open science activities might not be necessary (a few respondents shared this perspective in their answers).

Regarding the comment that our survey does not represent the opinions of scholars in general, but only a very small group already working on open science: as we stated in our previous response, we acknowledge that the final set of respondents consists primarily of persons close to the open science community. We initially hoped for a larger number of responses from various scholars, but open science practices are not yet well established in many research communities. Because of this, it might well be that the answers are actually more informative to guide our later RDA recommendations as they come from people who already have had thoughts about the issues. We highlighted this limitation in the text in lines 298-301.

*“Even though the survey was distributed to various research communities without the specific aim of targeting exclusively researchers engaged in OS, the final set of respondents were primarily individuals familiar with or active in OS, introducing a bias that may reflect the limited uptake of OS activities among many scholars”*.

3) Table 3 omits the number of respondents with “no opinion”.

> We think this is Figure 3 you are referring to. We edited the figure to include the percentages of “No opinion”. Thanks for spotting this!

4) The way Figure 4 is constructed is completely incomprehensible. It is derived from an open responses to an open questions. So it is impossible to understand the significance of the percentage. How many indications did each response contains on average? How many indications were derived from each questionnaire? If more than one indication is drawn from each questionnaire, how the percentage should be interpreted?

> We agree we didn’t provide enough details in the Methods to explain how Figure 4 was made. We now clarify this in lines 140-144. We have also updated the figure to make this more clear, including all the terms (and main categories) mentioned and coded as different types of rewards using our terminology.

*“Each respondent could have more than one preferred type of reward. To graphically represent the researchers’ preferences, we counted the frequency of a term being mentioned over the total number of respondents (230). For example, if the term “funding/grants for OS activities” was mentioned by 10 respondents, it would mean 4.3% of them preferred this type of reward.”*

5) Moreover, the question of the survey indicated some examples of possible responses that are precisely those we later find in the table, which makes one suspect a bias induced by the way the questionnaire was administered.

> We gave a few examples to clarify the open-ended question (Section 4 of the questionnaire) because the terminology for open science rewards is not yet well established, which may lead to confusion for numerous scholars. However, respondents still provided a variety of new categories along with the context explaining those categories. This feedback helped improve and update our initial terminology framework through data-driven coding of open-ended responses. Details can be found in the Methods section (lines 134-144).

6) The classification of disciplines is completely idiosyncratic (what are applied science or formal science?), adopting no established classification.

> We acknowledge that we didn’t provide enough details in the Methods to explain how the classification was made. For the re-submission of our manuscript we have completely re-classify disciplines following FORD, “OECD (2015), Frascati Manual 2015: Guidelines for Collecting and Reporting Data on Research and Experimental Development”. We now clarify this in lines 129-133.

*“The free text provided by respondents regarding their disciplines (they were asked to choose up to three main disciplinary fields) was classified into a unique field and subfield according to OECD [14] fields of research and development. One respondent did not indicate any discipline, while three others provided insufficient information to correctly classify their disciplinary field (e.g., “Multidisciplinary” and “Science”).”*

As we provided the entire dataset and workflow with the manuscript (see it at our GitHub repository <https://github.com/bienflorencia/rda-sharc-survey/> and Grattarola [20]), the original responses are available and can be reclassified using other systems.

# **Response to Reviewer #3**

1) In general, the significance of the sample is not made explicit: 230 responses out of the entire potential population of researchers are not significant. This is a major weakness of the proposed study. I suggest to explain what is the added value of this study more in detail.

> As we mentioned in our response #1 to the editor, our study was mainly qualitative and intended as a step to collect feedback to develop a set of recommendations to help implement various reward schemes at different levels (Mabile et al, 2024; doi: 10.15497/RDA/000117). As such, it did not aim at gathering a representative sample or at being statistically significant in the sense of a quantitative survey. Instead, we were interested in collecting different voices about recognition systems around the world. Since we were able to get responses from researchers from 37 countries (all continents), mostly familiar with open science, and only in a few of them we actually found rewarding schemes in place, we believe the sample can be considered sufficient for the purpose of identifying these rewarding schemes. We now explain in more detail in lines 309-315 what is the added value of our study.

*“Despite the survey sample not being representative, as a cross-cultural group of authors, we were able to crosswalk diverse perspectives on what rewards and incentives can mean in different countries and cultures. Thus, the findings of our study can contribute to OS theory and practice by suggesting OS rewards terminology and can inform a broad range of stakeholders, e.g., involved in research and innovation systems, in implementing OS rewarding schemes. In doing so, our work paves the way for broader, more robust quantitative studies in the future.”*

2) Furthermore, in the distribution of responses between countries (p. 6), the percentages of responses for France, US, South Korea, Uruguay and then "other countries" are mentioned. On page 9, it is then stated that positive responses regarding the existence of reward systems came from respondents from France, US, Netherlands, Finland, Slovenia, Spain, UK and Germany, while we have no idea how many responded from most of the countries mentioned here, making the figure even less significant. Moreover, it is not clear whether the respondents came from the same institutions or not. As they were asked to explicit their institutional policies, this information could be relevant.

> This is a good point. We now include more countries in the list, and mention as “other countries” those with only one response in lines 158-162:

*“We received 230 responses, with a survey’s completion rate of over 62%, from individuals across five continents covering 37 countries: 21.3% from France, 18.3% from the United States, 17.8% from South Korea, 5.2% from Uruguay, 4.8% from Argentina, 3.9% from Germany, 3.5% from United Kingdom, 3% from the Netherlands, 2.2% from Brazil, and 20% from other countries”.*

More details about the respondents’ countries, institutions, etc., can be found in the aggregated responses we provide openly in the S4 file.

3) In addition, the following 2 sentences in the abstract are in contradiction: "Despite most participants reporting that their organisation had one form or another of formal Open Science policies, the majority indicated that their organisation lacks any initiative or tool that provides specific credits or rewards for Open Science activities. For instance, researchers from France, the United States, the Netherlands and Finland affirmed having such mechanisms in place." I suggest to reformulate them to make your statement more clear.

> We agree this sentence needed reformulation, it now reads as follows (line 45):

*“However, researchers from France, the United States, the Netherlands and Finland affirmed having such mechanisms in place.”*
